# Editorial: Medical education in uncertain times: threats, challenges, and opportunities of COVID-19

**DOI:** 10.3389/fpsyg.2024.1467070

**Published:** 2024-08-28

**Authors:** Changiz Mohiyeddini, Stephen Francis Loftus

**Affiliations:** Oakland University William Beaumont School of Medicine, Rochester, MI, United States

**Keywords:** medical education, COVID-19 pandemic, Cognitive Load Theory, extraneous load, stress, healthcare workers

The World Health Organization declared “coronavirus disease 2019” (COVID-19) a pandemic on March 12, 2020. As of July 2024, the total number of confirmed COVID-19 cases worldwide exceeds 774 million, and the number of deaths attributed to the virus is over 7 million (World Health Organization, 2024). In addition to the human costs, the economic impact of COVID-19 has been profound and multifaceted. As a result of widespread lockdowns, supply chain disruptions, and a significant reduction in consumer and business spending, the International Monetary Fund (2021) estimated that the global economy contracted by ~3.5% in 2020 alone, amounting to 3.06 trillion US dollars. Additionally, the World Bank reported that the pandemic pushed an estimated 97 million people into extreme poverty in 2020, reversing decades of progress in poverty reduction (World Bank, 2021).

In addition to the human and economic consequences, both COVID-19 and the extraordinary measures to contain it have had, and continue to have, an enormous impact on higher education. Globally, the COVID-19 pandemic has forced academic institutions to adopt online education approaches, with significant and unknown implications for educators and learners. It is clear that some disciplines can smoothly adapt to online education. However, the difficulties that came with the COVID-19 pandemic, meant that medical education faced massive challenges due to its highly lab-based and hands-on structure.

Firstly, medical education had to acknowledge that most educational and psychological theories on learning and instructional design were developed for “normal circumstances.” However, “Learning and instructional procedures do not occur in a situational vacuum” (Taylor et al.). Important questions included which theories could be used to understand the implications of the new psychological environment under which educators and learners had to, and still have to, perform, and how these theories needed to be revised (Pauli et al., 2008; Loftus, 2015).

Secondly, and in a related vein, medical education had to consider that “there is no learner or educator without a past” (Taylor et al.). Because of variations in their pasts, learners always come to the educational experience with different strengths and weaknesses. Hence, medical education had to consider inter- and intraindividual differences in instructing, teaching, and learning during a pandemic. Thirdly, while acknowledging the negative impacts of the COVID-19 pandemic, medical educators had to look for and capitalize on opportunities that the pandemic offered to enhance the educational experience of students. Therefore, the aim of the Research Topic on “*Medical education in uncertain times: threats, challenges, and opportunities of COVID-19*” was to provide a platform for researchers to deliver sound theoretical approaches and empirical evidence to inform medical education, public education policies, to support and advise governments and policymakers in introducing sustainable, feasible, and cost-effective guidelines for medical education. In addition, moving from a deficit-oriented approach toward a positive psychology of trauma and loss, it aimed to encourage studies that address COVID-19 as a “chance” not only to improve medical education, health care systems, and fight health disparities, but also to reconceptualize what education for any professional practice, such as healthcare, should involve (Higgs et al., 2010).

Taylor et al. expanded on Cognitive Load Theory (CLT) by incorporating the psychological impact of COVID-19 on learning environments. The study adapted CLT principles to reduce extraneous load, facilitating students' educational activities in a virtual environment. This adaptation is crucial for medical education, where reducing extraneous load can help maintain productive learning despite the pandemic's constraints. There are lessons here for reducing extraneous load under normal circumstances that can improve the learning experience.

De Micheli et al.'s examination of psychological distress among Italian healthcare students highlights that the mental health challenges of students were aggravated by the pandemic. Their findings reveal that perceived control and concern for patients positively influenced students' readiness to combat the COVID-19 pandemic. This underscores the need for universities to implement robust mental health support systems that integrate both enhancing protective factors and mitigating debilitating conditions to help students navigate crises.

Hassan et al. assessed Egyptian clinical dental students' perceptions of online education during COVID-19. Their results show that that the academic performance of over 97% of students was negatively impacted by the pandemic. However, while theoretical education was rated neutrally, practical education was rated less effective. Reduced interaction with educators and poor internet connectivity were identified as significant barriers impeding students' learning. However, despite these challenges, students favored a hybrid approach, combining online and in-person education, suggesting a need for flexible learning models that can adapt to varying circumstances.

Mohiyeddini explored the multifaceted challenges and opportunities presented by the increasingly diverse cultural landscape in healthcare. He emphasizes the necessity for medical education to adapt and equip healthcare professionals with the skills to provide equitable care. Highlighting the growing impact of globalization, migration, and multicultural societies, he argues for comprehensive Cross-Cultural Medical Education to improve health outcomes, reduce disparities, and ensure that both patient and provider cultural backgrounds are effectively acknowledged and integrated into medical practice.

Soll et al. investigated the efficacy of an asynchronous blended learning model for teaching Cognitive Behavioral Therapy (CBT) to postgraduate health professionals in Germany during COVID-19. Their study compared online training with traditional in-person methods and found that online training was non-inferior regarding content delivery and didactic quality. Although there were minor benefits to in-person training in terms of professional development, the results support the integration of online elements in CBT education beyond the pandemic.

Klasen et al. conducted a qualitative investigation into the experiences of medical students working on the COVID-19 frontline. The study revealed a mix of relief, stress, and gratitude among students, who found a sense of purpose in their contributions despite the challenges. The findings emphasize the necessity for robust support systems to ensure students' mental health during crises.

Vallone et al. explored the impact of “technostress” on academic motivation and psychological health among European university students. The study identified that techno-overload, work-home conflict, and amotivation, had a direct negative impact on some students. For some students, however, techno-ease, techno-reliability, and intrinsic and extrinsic motivation had a direct protective role on their psychological health. These findings highlight the need for tailored interventions to balance technology use and enhance student wellbeing.

He and Li investigated death attitudes and anxiety among Chinese medical interns post-COVID-19. Their study found varying levels of death anxiety and acceptance influenced by personal and professional experiences with the pandemic. These findings underscore the importance of addressing death-related attitudes and providing psychological support to medical interns.

Taken together, these studies collectively demonstrate the profound and diverse impacts of the COVID-19 pandemic on health professions, education and mental health. They emphasize the necessity for innovative educational approaches, enhanced support systems, and targeted psychological interventions to ensure the continued effectiveness and wellbeing of (medical) students. Hence, it is imperative that medical education evolves to meet these new challenges, integrating lessons learned to improve future resilience and adaptability. It is widely accepted that societal adaptations to help the disabled end up benefiting everyone. The lessons learned from providing medical education in a pandemic can be used to improve medical education during normal times.
